# Circular to the Physicians of Tennessee and Kentucky

**Published:** 1851-08

**Authors:** 


The following Circular of the Committee on Epidemics, appointed by the American Medical Association at its recent meeting in Charleston, S. C., has been addressed to the physicians of Tennessee and Kentucky. We hope the requests of the committee will be attended to.

"CIRCULAR TO THE PHYSICIANS OF TENNESSEE AND KENTUCKY.

"*Georgetown, Ky.*, July 16, 1851.

"The American Medical Association, at its last meeting made it the duty of the undersigned to report to the next meeting, which is to be held in Richmond, Va., on the first day of May, 1852, upon the Epidemics of Tennessee and Kentucky.

"We most earnestly solicit the co-operation of our brethren of the two States, in furnishing the materials necessary to make this report. It is surely, unnecesssary to say one word upon the vast benefit to be conferred upon the community, and upon the profession, by a work of this kind, faithfully executed.

"Let no physician say, 'there are plenty of physicians to make these returns without my troubling myself about them.' No person can describe an epidemic so well as he who has been in its midst: nor can he be certain that the same epidemic does not vary in important features in different localities. It is earnestly hoped, that we will receive a return from every county in each State—even if no epidemic shall have appeared in a county, that fact is important to be known.

"It is desired, that the the returns made to us, embrace all epidemics which shall have occurred between the 1st of January and 31st of December, 1851, to be forwarded to one of us *immediately* after the last mentioned period.

"We desire *carefully observed facts*, upon any point connected with epidemics; but would suggest *particular* attention to the following heads:

"Causes supposed to have given rise to the epidemic.

"Causes which favored its spread.

"Causes which retarded its progress.

"Prophylactics.

"Age, sex, color, employment, diet, and habits as to exposure and temperance, of those most liable.

"The extent of its range.

"Prominent symptoms during its different stages.
"Proportional mortality—*Post-Mortem appearances.*
"Treatment.
"Medical Topography—including the nature of the soil and geological characters—character and average temperature of the springs and wells.
"Meteorological observations—mean, monthly, and annual temperature, weight and moisture of the atmosphere, etc., etc.
"Is it too much to request that you will fill up the annexed table,* so far as your own and your medical friends' practice will admit, and return it with your report?

"W. L. Sutton, M.D., Ch'mn, Georgetown, Ky.
"Theo. S. Bell, M.D., Louisville, Ky. } Committee.
"W. K. Bowling, M.D., Nashville, Tenn.

"We perceive that there is a movement on foot to organize a State Medical Society for Kentucky, will you give us your aid in forming it?"

* We are compelled to omit the table.

